# Lymphocyte antigen 6 complex locus G6D downregulation is a novel parameter for functional impairment of neutrophils in aged mice

**DOI:** 10.3389/fimmu.2022.1001179

**Published:** 2022-10-27

**Authors:** Suguru Saito, Alato Okuno, Toshio Maekawa, Ryoki Kobayashi, Osamu Yamashita, Noriyuki Tsujimura, Morihiko Inaba, Yasushi Kageyama, Noriko M. Tsuji

**Affiliations:** ^1^ Division of Cellular and Molecular Engineering, Department of Life Technology and Science, National Institute of Advanced Industrial Science and Technology (AIST), Tsukuba, Japan; ^2^ Department of Biomedical Sciences, Cedars-Sinai Medical Center, Los Angeles, CA, United States; ^3^ Department of Dentistry, Faculty of Medicine and Dentistry, University of Alberta, Edmonton, AB, Canada; ^4^ Department of Health and Nutrition, Faculty of Human Design, Shibata Gakuen University, Aomori, Japan; ^5^ Division of Immune Homeostasis, Department of Pathology and Microbiology, Nihon University School of Medicine, Tokyo, Japan; ^6^ Division of Microbiology, Department of Pathology and Microbiology, Nihon University School of Medicine, Tokyo, Japan; ^7^ iFoodMed Inc., Tsuchiura, Japan; ^8^ Division of Microbiology and Immunology, Department of Infection and Immunology, Nihon University School of Dentistry at Matsudo, Matsudo, Japan; ^9^ Technical Service Department, CLEA Japan, Inc., Tokyo, Japan; ^10^ Tokyo Animal and Diet Department, CLEA Japan, Inc., Tokyo, Japan; ^11^ Department of Food Science, Jumonji University, Niiza, Japan

**Keywords:** neutrophils, lymphocyte antigen 6 complex locus G6D (Ly-6G), anti-bacterial immunity, myeloid lineage cells, aging

## Abstract

Immunological aging is a critical event that causes serious functional impairment in the innate immune system. However, the identification markers and parameters are still poorly understood in immunological aging of myeloid lineage cells. Here, we show that a downregulation of lymphocyte antigen 6 complex locus G6D (Ly-6G) observed in aged mouse neutrophils could serve as a novel marker for the prediction of age-associated functional impairment in the neutrophils. Ly-6G expression was significantly downregulated in the bone marrow (BM) neutrophils of aged mice compared to young mice confirmed by flow cytometry analysis*. In vitro* experiments using BM-isolated neutrophils showed significant downregulations in their activities, such as phagocytosis, reactive oxygen species (ROS) production, interleukin (IL)-1β production, neutrophil extracellular trap (NET) formation, and migration as well as bacterial clearance, in the aged mouse neutrophils compared to those of young mice counterparts. Interestingly, the magnitudes of functional parameters were strongly correlated with the Ly-6G expression in the neutrophils. Thus, our results suggest that downregulation of Ly-6G reflects the age-associated functional attenuation of the neutrophils.

## Introduction

Aging is an unavoidable physiological event and affects various biological processes including immunity (1). Both the innate and adaptive immune systems are impaired by aging that the influences are widely recognized in our bodies with multiple consequences (1). The impact of aging on the immune system was firstly documented for adaptive immunity, specifically for T-lineage cells (2). The aged adaptive immune system impaired their responses resulting in overinflammatory conditions, increased susceptibility to infections, and loss of resistance ability against tumorigenesis (2). T cells in the aged environment are prone to a shift into senescence condition, and they upregulate the expression of co-inhibitory molecules, such as programmed cell death protein 1 (PD-1 also known as CD279), T-cell immunoglobulin and mucin-domain containing-3 (TIM-3 also known as CD366), and V-domain Ig suppressor of T-cell activation (VISTA) (2, 3). These changes cause impaired T-cell functioning which results in a disturbance of the whole immune system. Similarly, this trend of immune disturbance due to immune aging is also observed in the innate system. Dysfunctions of innate immune cells, such as neutrophils and macrophages, cause an increase in pathogenic invasion, chronic inflammation, and risk of atherosclerosis (4).

Neutrophils are frequently studied due to their central role in the innate immune system, specifically their involvement in antibacterial immunity (5). Under bacterial invasion, neutrophils function to eliminate the bacteria through several activities, such as phagocytosis, neutrophil extracellular trap (NET) formation, and reactive oxygen species (ROS) production (5–7). Thus, it is expected that the impairment of neutrophil functioning will result in the collapse of the primary defense against pathogen colonization. Moreover, it was illustrated that neutrophil function was altered through age-associated impairment of NETosis, which was induced by Toll-like receptor ligands and bacterial phagocytosis in the mouse model (8, 9). Furthermore, the neutrophils obtained from aged mice displayed less migration toward interleukin (IL)-8 (10). Human neutrophil activity characterized by an attenuated migration was also influenced by aging (11). However, knowledge in molecular mechanisms is still limited in age-associated dysfunction of neutrophils. A specific marker which can be used for the prediction of the functional impairment is also unknown in neutrophils; otherwise, a complex *in vitro* experimental system is required to assess the function.

Neutrophils are generally identified by highly expressed lymphocyte antigen 6 complex locus G6D (Ly-6G) in myeloid lineage cells determined by CD11b expression in mice (12). Alternatively, CD66b are specific surface markers on human neutrophils (13). Ly-6G is a 21–25-kD glycosylphosphatidylinositol (GPI)-linked cell surface protein which belongs to the Ly-6 family and is identified as a differentiation antigen expressed on mature neutrophils in both bone marrow (BM) and the periphery (14). In humans, CD177 was identified as a homolog of Ly-6G in mice (12). After the identification of Ly-6G in 1993, the physiological function was unknown for many years. Recently, some findings have been reported on the role of Ly-6G in neutrophils. A report showed that Ly-6G interacted with β2 integrins and regulated their migration. To explain this phenomenon, the authors provided evidence that β2 integrins CD11a and CD11b were colocalized with Ly-6G on the neutrophils and orchestrated with each other in the migration process. Furthermore, the ligation of Ly-6G by using monoclonal antibody (mAb, 1A8) suppressed the migration (15). Another report showed that Ly-6G deficiency critically impaired the early recruitment of neutrophils in an *Leishmania major*-infected site (16). In this report, the role of Ly-6G in the differentiation step was also discussed. Although Ly-6G is considered as a differentiation marker, Ly-6G-knockout (KO) mice did not show any critical impacts on the development of neutrophils (16). Thus, the function of Ly-6G is still controversial and has not been understood yet.

In this report, we show that Ly-6G expression is downregulated on the neutrophils originating from aged mice compared to those of young mice. Furthermore, we found a strong correlation between the expression level of Ly-6G and the grade of immunological parameters in antibacterial immunity, which are attenuated by aging, in the neutrophils. Thus, we propose that Ly-6G downregulation is a novel marker for age-associated functional impairment of neutrophils.

## Materials and methods

### Mice

Both young and aged C57BL/6J mice were obtained from CLEA Japan, Inc. (Tokyo, Japan). The mice were maintained in a specific pathogen-free (SPF) condition with 12-h day/night cycles and were allowed free access to food and water. The mice were prepared for young (10 to 20 weeks) and aged (20 to 22 months) groups. Since we had experienced the difficulty to keep enough number of aged male mice in our colony, we decided to use female mice in this study. All aged mice were screened for their condition prior to use for experiments, and the mice suspected to have abnormalities (e.g., tumor-bearing) were excluded from the subject. The data of body weight (g), spleen weight (mg), and body temperature (°C) were shown for all mice, as shown in **Supplemental Figure 1**. All experiment protocols were reviewed and approved by the Animal Welfare Committee of AIST (protocol No. 109) and CLEA Japan, Inc. (AE2157-121).

### Reagents and antibodies

Phorbol 12-myristate 13-acetate (PMA) was purchased from Merck/Millipore Sigma (Burlington, MA, USA). Lipopolysaccharide (LPS) and polyinosinic:polycytidylic acid (poly(I:C)) were purchased from *In vivo*Gen (San Diego, CA, USA). Fluorescein isothiocyanate (FITC)-labeled *E. coli* (K-12 strain) BioParticles, 2′,7′-dichlorodihydrofluorescein diacetate (H2DCFDA), carboxyfluorescein succinimidyl ester (CFSE), and SYTOX™ Green were purchased from Thermo Fisher Scientific (Waltham, MN, USA). Anti-CD45 (30-F11), anti-CD11b (M1/70), anti-Ly-6G (1A8), anri-Ly-6C (HK1.4), anti-CD18 (M18/2),anti-CD11a (M17/4), anti-CD16/CD32 (2.4G2), anti-CD64 (S18017D), anti-CD21/CD35 (7E9), anti-CXC chemokine receptors 2 (CXCR2) (SA044G4), anti-CXCR4 (12G5), and 7-amino-actinomycin D (7-AAD) were all purchased from BioLegend (San Diego, CA, USA). Recombinant mouse C–X–C motif chemokine 1 (CXCL1) and C–X–C motif chemokine 5 (CXCL5) were purchased from PeproTech (Cranbury, NJ, USA).

### Preparation of bone marrow neutrophils

The neutrophils were isolated from the bone marrow (BM) by the following protocol described in the previous reports (17, 18). Briefly, the BM cells were flushed out from the tibia and femur in RPMI complete medium (RPMI 1640 supplemented with 10% fetal bovine serum (FBS), penicillin 100 U/ml, streptomycin 100 U/ml) using a 10-ml syringe with a 27G needle. The BM cells were washed, and contaminated red blood cells (RBCs) were eliminated with RBC lysis buffer (Thermo Fisher Scientific). The neutrophils were magnetically enriched by using Neutrophil Isolation Kit, mouse (Miltenyi Biotec, Bergisch Gladbach, North Rhine-Westphalia, Germany), following the manufacturer’s instruction. The purified neutrophil samples with CD45+CD11b+Ly-6G+ >90% were used for experiments.

### Culture of bacteria

*Escherichia coli* (DH5α) was purchased from the American Type Culture Collection (ATCC). The frozen stock was thawed on ice, and the bacteria were cultured in LB medium following the product manual. The bacterial colony-forming unit (CFU/mL) was calculated by following the previous reports (17, 18).

### Phagocytosis assay

Neutrophils (1.0 × 10^7^/ml) were seeded in a 96-well plate (round bottom) in RPMI complete medium. The samples were treated with K-12 BioParticles (10 μg/ml) at 37°C for 60 min. The phagocytosis activity was measured by flow cytometry.

### ROS production assay

Neutrophils (1.0 × 10^7^/ml) were seeded in a 96-well plate (round bottom) in RPMI complete medium containing DCFDA (500 nM). The samples were treated with LPS (1 μg/ml), live *E. coli* (1.0 × 10^7^ CFU/ml), or vehicle control (PBS) at 37°C for 60 min without direct exposure to light. After incubation, ROS production was analyzed by flow cytometry.

### Cytokine production assay

Neutrophils (1.0 × 10^7^/ml) were seeded in a 96-well plate (flat bottom) and then were stimulated with LPS (1 μg/ml), poly(I:C) (10 μg/ml), or live *E. coli* (1.0 × 10^7^ CFU/ml). The control samples were treated with vehicle control (PBS). The cultures were incubated at 37°C overnight, and then the plate was immediately frozen at -80°C until use. For measuring the IL-1β concentration in the cultured medium, the plate was centrifuged at 300 g for 5 min and supernatant was collected for using enzyme-linked immunosorbent assay (ELISA).

### Migration assay

BM-isolated neutrophils were labeled with CFSE (5 μM) in PBS at 37°C for 15 min, then washed with PBS. The CFSE-labeled neutrophils (1.0 × 10^6^/ml) were seeded in a transmigration well (pore size 3 μm, Corning, NY, USA) in RPMI 1640 medium. The wells were placed on a 96-well plate which contains RPMI 1640 medium supplemented with CXCL1 (1 μg/ml) or CXCL5 (1 μg/ml), and then the plate was incubated at 37°C for 2 h. After the incubation, the fluorescence originated from the migrated cells was measured in the plate by a microplate reader (Spark, Tecan, Männedorf, Switzerland).

### NETosis assay

NETosis activity was assessed by following a method described in the previous report (19). Neutrophils (5.0 × 10^5^/ml) were seeded in a 96-well plate (flat bottom, poly-L-lysine coated) in RPMI complete medium containing PMA (50 nM) and incubated at 37°C for 4 h. After incubation, the cultured medium was collected for cell-free DNA (CFD), citrullinated Histone H3 (CitH3), and myeloperoxidase (MPO)–DNA complex assays, and the cells were used for NET staining. For the CFD assay, the released DNA in the cultured medium was stained with staging with SYTOX™ Green (500 nM) at 37°C for 15 min. CitH3 and MPO–DNA complex assays were performed by using the Citrullinated Histone H3 ELISA Kit (Cayman Chemical, Ann Arbor, MI, USA) and laboratory homemade ELISA system, respectively. The fluorescence and absorbance were measured by a microplate reader (Spark, Tecan). For NET staining, the cells were treated with PBS containing SYTOX™ Green (500 nM) at 37°C for 15 min followed by washing with PBS and fixation with 1% PFA. NETosis was observed using a fluorescence microscope (BZ-X700, Keyence, Osaka, Japan).

### Bacterial killing assay

Neutrophils (1.0 × 10^7^/ml) were seeded in a 96-well plate (flat bottom) in RPMI complete medium. The samples were exposed to live *E. coli* (1.0 × 10^7^ CFU/ml) at 37°C for 6 h. The cultured medium was harvested and diluted with PBS prior to seeding on an LB agar plate. The bacterial CFU was calculated in each sample. The efficiency of bacterial clearance was calculated by the following formula: Bacteria clearance (%) = *E. coli-*CFU/mL after incubation/Loaded *E. coli-*CFU/mL × 100.

### Flow cytometry

Cell surface markers, bacterial phagocytosis, and ROS production were analyzed using flow cytometers (LSRFortessa SORP and FACS Aria I; BD Biosciences, Franklin Lakes, NJ, USA). For the surface marker staining, the cells were first incubated with an FcR blocker (anti-CD16/CD32) at 4°C for 10 min followed by incubation with the antibodies in PBS/2% FBS at 4°C for 30 min. All data were analyzed by FACSDiva (BD Biosciences) and FlowJo (BD Biosciences). All flow cytometry analyses were performed by following a gating strategy shown in **Supplemental Figure 2**.

### Enzyme-linked immunosorbent assay

The IL-1β concentration in cultured medium was measured using the mouse IL-1β DuoSet ELISA Kit (R&D Systems, Minneapolis, MN, USA). All procedures were performed by following the manufacturer’s instruction.

### Statistics

Statistical analyses were performed by using GraphPad Prism (GraphPad Software, San Diego, CA, USA). Student’s *t*-test was used to analyze the data for significant differences.

## Results

### Comparison of the myeloid lineage population in BM of young and aged mice

**Figure 1:**
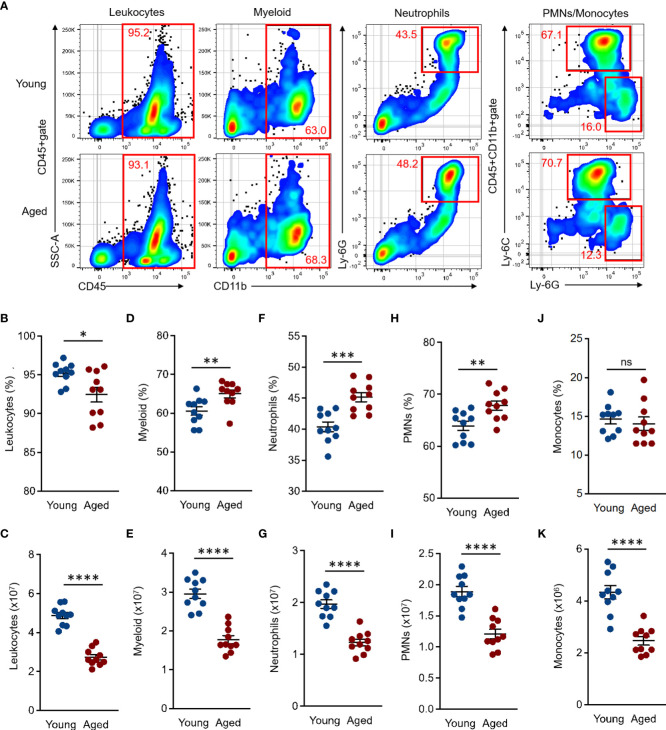
Comparison of myeloid lineage cells in BM of young and aged mice BM cells were isolated from the tibia and femur of both young (10 to 20 weeks) and aged (20 to 22 months) mice for flow cytometry analysis. Flow cytometry analysis was performed by following the gating strategy represented in **Supplemental Figure 2**. **(A)** Representative plots of total leukocytes, myeloid, granulocytes, PMNs, and monocytes in flow cytometry analysis. **(B, C)** Percentage **(B)** and number **(C)** of total leukocytes (CD45+). **(D, E)** Percentage **(D)** and number **(E)** of total myeloid (CD11b+ in CD45+gate). **(F, G)** Percentage **(F)** and number **(G)** of granulocytes (CD11b+Ly-6G+ in CD45+gate). **(H, I)** Percentage **(H)** and number **(I)** of PMNs (Ly-6G+Ly-6Cdim/+ in CD45+CD11b+gate). **(J–K)** Percentage **(J)** and number **(K)** of monocytes (Ly-6G-Ly-6Chi in CD45+CD11b+gate). The number of cells in the populations were calculated as per mouse (BM). The cumulative data are shown as mean ± SEM values of 10 samples. Student’s *t*-test was used to analyze data for significant differences. Asterisks indicate significance: *p < 0.05, **p < 0.01, ***p < 0.001, and ****p < 0.0001. ns; not significant.

The BM is a major site of innate immune cell differentiation and proliferation. Moreover, the immune system is constantly subjected to various factors, such as physiological condition, disease, the microbiome, and aging. These factors subsequently alter the phenotype of immune cells produced in the BM (20, 21). To further understand the age-associated impact in the steady state, we first characterized and compared the myeloid lineage cells in the BM between young and aged mice. The total numbers of BM cells were significantly decreased in aged mice compared to young mice (**Supplemental Figure 1D**). The frequency and number of leukocytes (CD45+) were also decreased in aged mouse BM as compared to those of young mice (Percentage (%): Young 95.21 ± 0.27 SEM *vs*. Aged 92.46 ± 0.6 SEM, Number (×10^7^): Young 4.87 ± 0.1 SEM *vs*. Aged 2.73 ± 0.09 SEM) (**Figures 1A–C**). The percentages of total myeloid (CD11b+ in CD45+gate, Young 60.59 ± 0.7 SEM *vs*. Aged 65 ± 0.65 SEM), neutrophils (CD11b+Ly-6G+ in CD45+gate, Young 40.38 ± 0.5 SEM *vs*. Aged 45.16 ± 0.47 SEM), and polymorphonuclear leukocytes (PMNs, Ly-6G+Ly-6Cdim/+ in CD45+CD11b+gate, Young 63.89 ± 0.57 SEM *vs*. Aged 67.83 ± 0.56 SEM) were all significantly increased in aged mice compared to the counterparts of young mice, while the cell numbers were decreased in those populations affected by decreased total BM leukocyte (CD45+) cells in the aged mice (Myeloid (×10^7^): Young 2.96 ± 0.08 SEM *vs*. Aged 1.78 ± 0.07 SEM, Neutrophils (×10^7^): Young 1.97 ± 0.05 SEM *vs*. Aged 1.23 ± 0.04 SEM, PMNs (×10^7^): Young 1.89 ± 0.05 SEM *vs*. Aged 1.21 ± 0.05 SEM) (**Figures 1A, D–I** ). The percentages of monocytes (Ly-6G-Ly-6Chi in CD45+CD11b+gate) were comparable between young and aged mice (Percentage (%): Young 14.62 ± 0.38 SEM *vs*. Aged 14.04 ± 0.55 SEM), while the cell numbers were decreased in aged mice similar to other subsets (Numbers (×10^6^): Young 4.33 ± 0.16 SEM *vs*. Aged 2.46 ± 0.1 SEM) (**Figures 1A, J, K**).

Taken together, the myeloid population in aged mouse BM has a specific tendency which is characterized by an increased frequency of neutrophils and decreased numbers of all myeloid lineage cells.

### Expression levels of cell surface marks are downregulated in the myeloid lineage cells of aged mice

**Figure 2:**
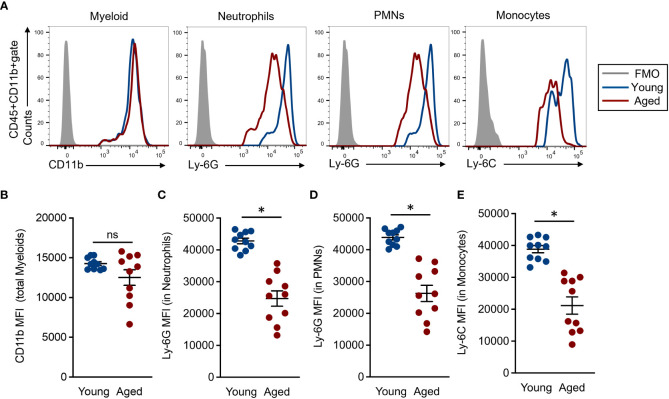
Comparison of common myeloid marker expression in each cell population **(A)** Representative histograms in flow cytometry analysis. Each population was determined by following the gating strategy represented in **Supplemental Figure 2**. **(B)** CD11b MFIs in the total myeloid population. **(C-D)** Ly-6G MFIs in granulocyte **(C)** and PMN **(D)** populations, respectively. **(E)** Ly-6C MFIs in the monocyte population. The cumulative data are shown as mean ± SEM values of 10 samples. Student’s *t*-test was used to analyze data for significant differences. Asterisk indicates significance: *p < 0.01. ns; not significant.

Myeloid lineage cells, such as neutrophils and monocytes, were characterized by the expression of specific cell surface markers named Ly-6G and Ly-6C, respectively (22). The expression intensities of these molecules are generally strong, clear, and distinct from other markers; therefore, these are commonly used in the identification of the cells. However, there are very few studies looking into the expression levels of Ly-6G and Ly-6C on neutrophils and monocytes in aged mice. Since we were interested in the expression differences of myeloid lineage-specific markers on BM myeloid lineage cells of young and aged mice, we compared the expression intensities of CD11b, Ly-6G, and Ly-6C in total myeloid, neutrophils, and monocytes, respectively. The CD11b expression level was comparable between young and aged mice in the total myeloid population (**Figures 2A, B**). In fact, some aged mice showed downregulated CD11b mean fluorescence intensities (MFIs) compared to those of young mice; however, these differences were not significant. Alternatively, the Ly-6G expression was significantly downregulated on neutrophil and PMN populations of aged mice as compared to those of young mice (**Figures 2A, C, D**). In addition, Ly-6C expression was also significantly downregulated in the aged mouse monocytes compared to young mice (**Figures 2A, E**). We also analyzed the expression levels of CD11b, Ly-6G, and Ly-6C on the myeloid cells in peripheral blood. The MFIs of Ly-6G and Ly-6C were both significantly downregulated in the aged mouse neutrophils and monocytes, respectively, compared to those of young mice. These results had constituencies to those observed in BM. In contrast, the CD11b expression level was comparable in total myeloid cells between young and aged mice (**Supplemental Figure 3**). Thus, the expression levels of specific markers, especially Ly-6G and Ly-6C, are down regulated in the myeloid lineage cells by aging.

### Functional impairment of neutrophils in aged mice

The immune system is altered by aging and is characterized by the promotion of disease progression and increases the risk of infectious diseases in humans (6). Similar conditions have been reported in the mice through the comparison of young and aged mice (23, 24). Adaptive immunity, especially for T-cell-based events, have been investigated well in the aging-related phenotype and function; however, further investigation is still required for innate immunity in the aged environment. Therefore, we decided to focus on the neutrophil’s function in aged mice. To clarify the age-associated functional change of neutrophils, we assessed some parameters related with antibacterial immunity by *in vitro* functional assays (7, 18, 19).

**Figure 3:**
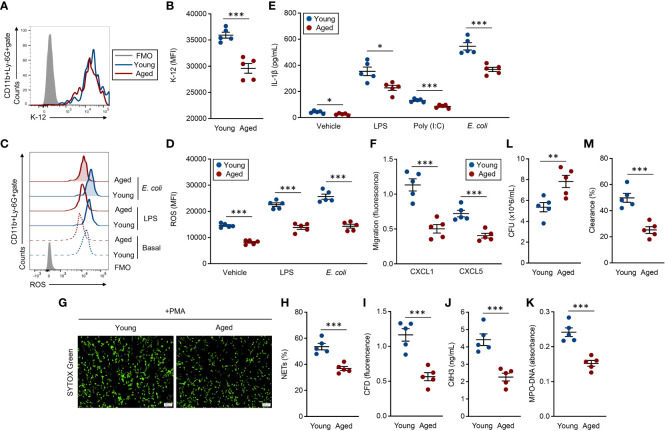
Functional impairment of aged mouse neutrophils **(A, B)** Phagocytosis assay against the *E. coli* K-12 strain. Neutrophils (1.0 × 10^7^/ml) were incubated with *E. coli* K-12 BioParticles (FITC labeled, 10 μg/ml) at 37°C for 2 h. The signal of the incorporated K-12 was detected by flow cytometry. **(A)** Representative histogram of incorporated K-12 signals. **(B)** Cumulative MFI values of the K-12 signals. **(C, D)** ROS production assay. Neutrophils (1.0 × 106/ml) were stimulated with LPS (1 μg/ml) or live *E* coli (1.0 × 10^6^ CFU/ml) at 37°C for 60 min. To detect the basal ROS level, fresh neutrophils were also subjected to the analysis. The intracellular ROS was stained with DCFDA (500 nM) and the signal was detected by flow cytometry. **(C)** Representative histograms of ROS. **(D)** Cumulative MFI values of ROS. **(E)** IL-1β production assay. Neutrophils (1.0 × 10^7^/ml) were treated with vehicle control (PBS), LPS (1 μg/ml), poly(I:C) (10 μg/ml), or live *E. coli* (1.0 × 10^7^ CFU/ml) at 37°C for overnight. The IL-1β concentration in the cultured medium was measured by ELISA. **(F)** Migration assay. CFSE-labeled neutrophils (1.0 × 10^6^/ml) were seeded in the transmigration well, then the cell migration towered to CXCL1 (1 μg/ml) or CXCL5 (1 μg/ml) was measured by the microplate reader after incubation at 37°C for 2 h. **(G–K)** NETosis assay. Neutrophils (5.0 × 10^5^/ml) were stimulated with PMA (50 nM) at 37°C for 4 h. The cells were stained with SYTOX× Green (500 nM) and observed by fluorescence microscopy **(G)**, and the percentages of NET-formed cells were calculated **(H)**. The CFD concentration in the culture medium was measured by SYTOX™ Green staining (500 nM) **(I)**. CitH3 concentration **(J)** and MPO-DNA **(K)** amount (absorbance) were measured by ELISA. **(L, M)** Bacterial killing assay. Neutrophils (1.0 × 10^7^/ml) were mixed with live *E coli* (1.0 × 10^7^ CFU/ml) at 37°C for 6 h. Bacterial CFU **(L)** and bacterial clearance **(M)** in the cultures were measured. The cumulative data are shown as mean ± SEM values of five samples. Student’s *t*-test was used to analyze data for significant differences. Asterisks indicate significance: *p < 0.05, **p < 0.01, and ***p < 0.001.

BM-isolated neutrophils were used for these assays, and the functional grades were compared between young and aged mouse neutrophils. Phagocytic activities against *E. coli* were significantly attenuated in aged mouse neutrophils compared to young mouse counterparts (**Figures 3A, B**). ROS production, which was induced by exposure with LPS and live *E. coli*, was also significantly downregulated in the aged mouse neutrophils as compared to young mouse counterparts. Interestingly, the basal production level of ROS was already suppressed in the aged mouse neutrophils (**Figures 3C, D**). Cytokine production was measured in both unstimulated and stimulated conditions in the neutrophils. IL-1β production was decreased in aged mouse neutrophils even at the basal level, and the differences compared to young mouse neutrophils were more obvious when the cells were stimulated with TLR ligands and live *E. coli* (**Figure 3E**). The migration ability toward CXCL1 and CXCL5 was impaired in aged mouse neutrophils compared with those of young mice (**Figure 3F**).

We also analyzed surface molecules, such as CD18, CD11a, CD64, CD16/CD32, CD21/CD35, CXCR2, and CXCR4, which have important roles in the functions of neutrophils (7). The expression levels of these integrins, Fc receptors, complement receptors, and chemokine receptors were all comparable between young and aged mouse neutrophils (**Supplemental Figure 4**).

NETosis, a substantial event in the bacterial clearance, was induced by PMA treatment (7, 19). The NET formation was clearly attenuated in aged mouse neutrophils as compared to those of young mice under microscopic observation (**Figure 3G**). The frequency of NETosis cells was significantly decreased in aged mouse culture compared to that of young mice (**Figure 3H**). Further, correlated with the frequency of NETosis, the levels of released-CFD, citH3, and MPO–DNA complex were also significantly decreased in the aged mouse cultures as compared to the cultures originating from young mice (**Figures 3I–K**). Finally, we compared the bacterial clearance against live *E. coli* in young and aged mouse neutrophils. The bacterial CFU originating from the survival bacteria was larger in aged mouse culture than that of young mice (**Figure 3L**). In addition, bacterial clearance was promoted in young mouse culture compared to aged mouse culture (**Figure 3M**).

We investigate the morphological aspects between young and aged mouse neutrophils. The cell adhesion ability, which was confirmed by phalloidin green staining, was comparable between the neutrophils originating from young mice and those from aged mice (**Supplemental Figures 4A–B**). The size and granularity of the neutrophils were also similar between these two groups of mice (**Supplemental Figures 4C–E**).

Since Ly-6C was downregulated in the aged mouse monocytes, we compared the function of monocytes between young and aged mice. In phagocytosis and cytokine production assay, the results were comparable between young and aged mice (**Supplemental Figures 5A, B**).

Thus, the activities in antibacterial immunity are uniformly attenuated in the aged mouse neutrophils, leading to failed elimination of the bacteria.

### Ly-6G downregulation is a marker for functional impairment in aged mouse neutrophils

As shown in **Figure 2**, we found a significant downregulation of Ly-6G on aged mouse neutrophils compared to those of young mice. To address the biological mean of this alteration, we finally investigated the correlations between Ly-6G expression level and the parameters associated with the grade of antibacterial immunity of the neutrophils, as shown in **Figure 3**.

**Figure 4:**
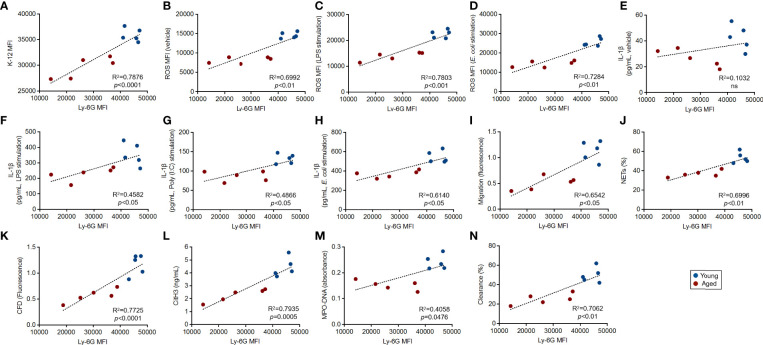
Correlation between Ly-6G expression and the parameters of antibacterial activities in the neutrophils Correlations between Ly-6G MFI and parameter values gained by functional assays. The linear regression graphs show correlations of Ly-6G MFIs *vs*. the values of incorporated K-12 MFIs **(A)**, basal ROS MFIs **(B)**, ROS MFIs in LPS **(C)**, live *E. coli* stimulation **(D)**, IL-1β concentrations in vehicle treated **(E)**, LPS **(F)**, poly(I:C) **(G)**, live *E. coli* stimulation **(H)**, migration **(I)**, parentage of NETosis **(J)**, CFD concentrations **(K)**, CitH3 concentration **(L)**, MPO-DNA amount (absorbance) **(M),** and percentages of bacterial clearance **(N)**, respectively. R^2^ and *p*-values were calculated by using GraphPad Prism.

Interestingly, all of the immunological events, except basal IL-1β production level, showed strong correlations with Ly-6G expression levels on the neutrophils. Furthermore, we found that the plots that originated from young mice were placed in the limited areas; however, the plots of aged mice were distributed especially by following Ly-6G expression levels in the linear regressions (**Figures 4A–N**).

We also investigated whether Ly-6C expression had a strong correlation with functional parameters. Different from Ly-6G in neutrophils, Ly-6C downregulation was not a critical factor in the functional impairment of monocytes, because there were no significant correlations between Ly-6C expression level and the values of functional parameters (**Supplemental Figure 5C–E**).

Thus, Ly-6G downregulation is tightly correlated with the functional impairment of the neutrophils in aged mice.

## Discussion

Concurring with our findings as well as previous reports, neutrophils are obviously dysfunctional in the aged environment in mice (**Figure 3**). This comprehensive dysfunction is a predictable event in humans as well. The capability to easily assess the functional level and possibly enhance the function of neutrophils would serve as a great help to maintain neutrophil-basis innate immunity in aged individuals. This is the ultimate goal of our concept which is similar to the concept of preventive medicine. Currently, we can only assess the function of neutrophils through *in vitro* studies. In animals, we can perform *in vitro* experiments using isolated neutrophils; however, there are several issues in the case of humans, such as sample collections and ethical statements. Thus, our finding of the correlation between Ly-6G expression level and functional grade on the neutrophils will be a useful marker for estimation of the age-associated attenuation of neutrophil function (**Figures 3**, **4**).

However, there is a critical difference between humans and mice, because Ly-6G is not expressed on human neutrophils. A homolog of mouse Ly-6G is CD177 in humans; however, the participation of this molecule in immune function of neutrophils is unknown. Alternatively, CD66b could be corresponding molecules to Ly-6G by reason of specific expression pattern on mature human neutrophils (12). We have not tried to identify the age-associated downregulated molecules, which correspond to mouse Ly-6G, in human neutrophils yet. It must be performed to confirm the consistency of current findings between humans and mice.

We also did not investigate the molecular mechanism in Ly-6G downregulation on the aged mouse neutrophils. In fact, the downstream signaling of Ly-6G has not been identified yet, and the characterization has never been performed well so far in our understanding. To reveal the mechanism of Ly-6G-associated functional regulation in neutrophils, we must perform a wide investigation of genetics and molecular biology bases. We wonder whether the Ly-6G downregulation is a trigger or just a consequence of the functional impairment of the aged mouse neutrophils. Because of the structural feature of Ly-6G, as a GPI-linked cell surface protein, it is not smooth to consider the signal transduction in the cytosol consequently attenuating the cell function (12–14). In this understanding, the downregulation might be a physiological consequence of the aging of the neutrophils. Interestingly, other myeloid-lineage markers, such as CD11b and Ly-6C, were also downregulated on the total myeloid and monocytes in aged mice (**Figures 2A, B, E**). This implies that these markers as well as Ly-6G are comprehensively downregulated on the myeloid lineage cells in aged mice. We were interested in the possibility whether Ly-6C could be a marker for functional dysregulation in monocytes and monocyte-lineage-originated immune cells; however, there was no significant correlation between Ly-6C downregulation and functional impairment in monocytes (**Supplemental Figures 5A–E**).

Our study has some limitations. First thing is the variation of the mice in their age. We compared young (10–20 weeks) and elementally aged mice (20–22 months); therefore, we also have to investigate Ly-6G expression and the functions of the neutrophils at the intermediate age (e.g., 12 months). This is an important point to understand when the Ly-6G downregulation becomes obvious, and the expression level starts to show a strong correlation with the age-associated dysfunction of the neutrophils. In addition, this study used female mice. It has been introduced gender difference-based functional alterations in neutrophils (25, 26); therefore, we have to consider the difference between male and female in our current finding. Next, we are not able to explain any possible reasons in the representing age-associated Ly-6G downregulation and the cause of functional impairment in the aged mouse neutrophils. One of the considerable causes in Ly-6G downregulation in the aged mouse neutrophils is failure of the differentiation and maturation step. In general, Ly-6G is highly expressed in mature neutrophils; however, the expression is relatively lower in the immature neutrophils in mice (27). It is well known that BM-leaked immature neutrophils are frequently observed under severe inflammatory conditions originating from pathogen invasion, and these cells showed less activity compared to mature neutrophils in human cases (28–30). Although there is no solid evidence that the aged environment leaks the immature neutrophils, we suspected that it is a possible response to compensate for weakening neutrophil-based innate immunity in the aged environment.

There are still some required points to reveal the details in our findings; however, we try to propose that Ly-6G downregulation is a novel marker for the prediction of age-associated functional impairment in mouse neutrophils.

## Data availability statement

The original contributions presented in the study are included in the article/**Supplementary Material**. Further inquiries can be directed to the corresponding authors.

## Ethics statement

This study was reviewed and approved by Advanced Industrial Science and Technology (AIST, protocol No. 109) and CLEA Japan, Inc. (AE2157-121).

## Author contributions

SS and NMT established the concept in this study. SS, AO, TM, RK, OY, NT and MI performed experiments. SS established analysis methodologies and performed data analyses. NMT, OY, NT, MI and YK had responsibility in animal resources. SS wrote the original manuscript. SS and NMT reviewed and finalized the manuscript. All authors participated in the discussion. SS and NMT supervised this study. All authors contributed to the article and approved the submitted version.

## Funding

This study was supported by the AIST- CLEA-Japan Joint Research Fund (NMT, YK), Japan Society for the Promotion of Science (21K15958 (SS)), Japan Society for the Promotion of Science (19H04042 (NMT)), and Mishima-Kaiun Memorial Fund (SS). The funder was not involved in the study design, collection, analysis, interpretation of data, the writing of this article or the decision to submit it for publication.

## Acknowledgments

We thank Yasaman Bahojb Habibyan M.sc and Maria Alexiou Ph.D for providing support in manuscript writing. We also thank Dr. Yohichi Shimma for providing a support in project administration.

## Conflict of interest

OY, NT, MI and YK are employees in CLEA Japan, Inc. TM is an employee in iFoodMed, Inc. RK and NMT are scientific advisors in iFoodMed, Inc.

The remaining authors declare that the research was conducted in the absence of any commercial or financial relationships that could be construed as a potential conflict of interest.

## Publisher’s note

All claims expressed in this article are solely those of the authors and do not necessarily represent those of their affiliated organizations, or those of the publisher, the editors and the reviewers. Any product that may be evaluated in this article, or claim that may be made by its manufacturer, is not guaranteed or endorsed by the publisher.
