# Supplementary material for: Lymphocyte antigen 6 complex locus G6D downregulation is a novel parameter for functional impairment of neutrophils in aged mice
Source: Front Immunol. 2022 Oct 27;13:1001179. doi: 10.3389/fimmu.2022.1001179 (PMC9647080; doi:10.3389/fimmu.2022.1001179)
Supplement: Supplementary file 1 [file DataSheet_1.docx]

Supplementary Material

## Supplementary Figures


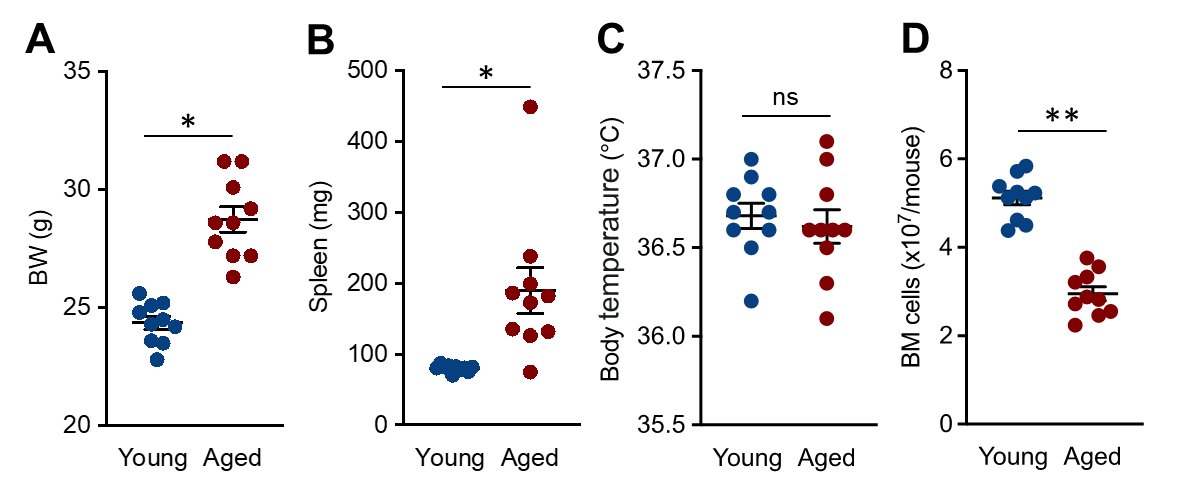


**Supplemental figure 1. Fundamental information of mice used in this study**

BW (A), spleen weight (B), body temperature (C), and total BM cells (D) were measured in the mice before sacrificed for sampling. The cumulative data are shown as mean ± SEM values of ten samples. Student's *t*-test was used to analyze data for significant differences. Asterisk indicates significance: **p* < 0.01 and ***p* < 0.01. ns; not significant.


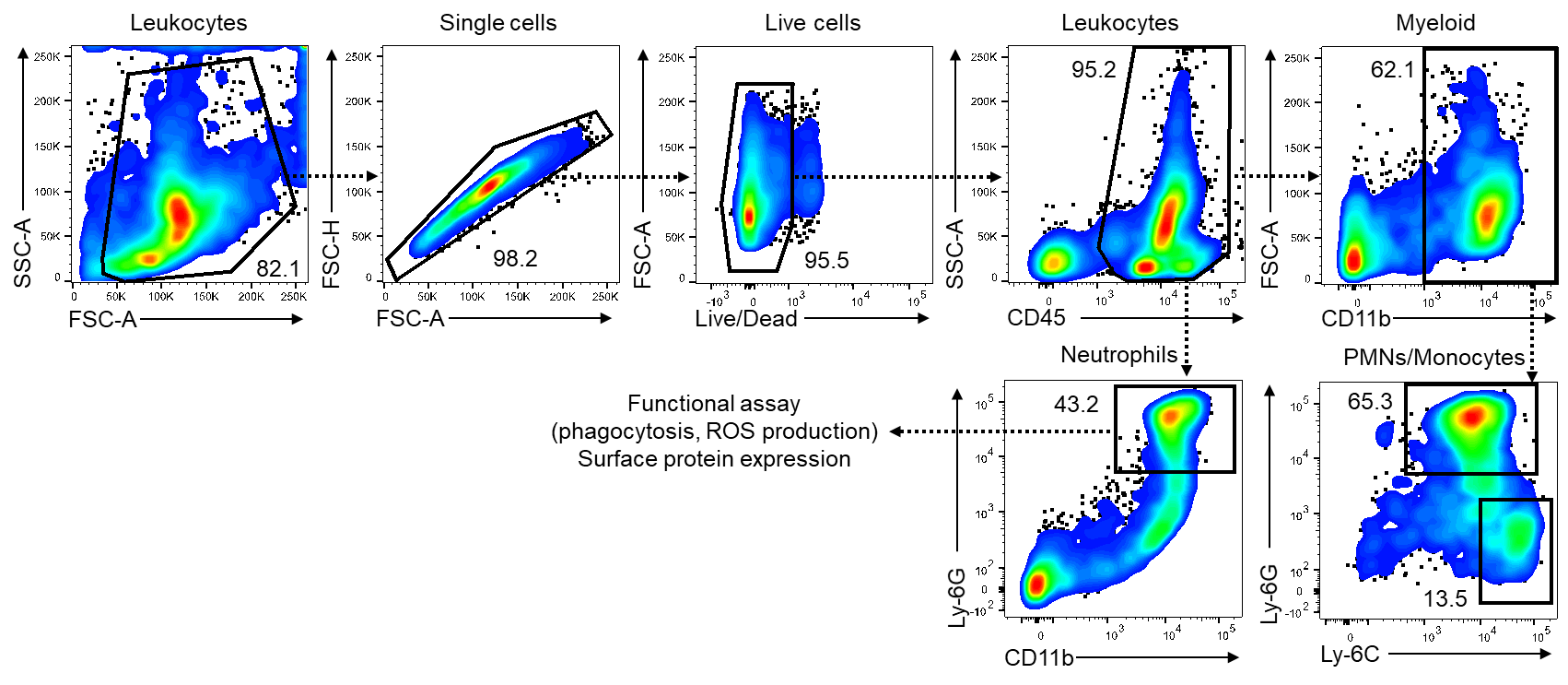


**Supplemental figure 2. Gating strategy for myeloid lineage cells characterization**

Leukocyte population was first gated, then Live population (7-AAD-) in single cells was selected to identify each myeloid lineage cells. Neutrophils (CD11b+Ly-6G+) were identified in CD45+gate. Myeloid population (CD11b+) was identified in CD45+gate, and PMNs (Ly-6G+Ly-6Cdim/+) and monocytes (Ly-6G-Ly-6Chi) were identified in myeloid gate.

**Supplemental figure 3. Expression intensity of surface markers in PB myeloid lineage cells**

PB was collected from cervical vein and the leukocytes were used for analysis after elimination of RBC. The expression of CD11b in total myeloid (A), Ly-6G in neutrophils (B) and Ly-6C in monocytes (C) were analyzed by flowcytometry. The cumulative data are shown as mean ± SEM values of five samples. Student's *t*-test was used to analyze data for significant differences. Asterisk indicates significance: **p* < 0.01. ns; not significant.


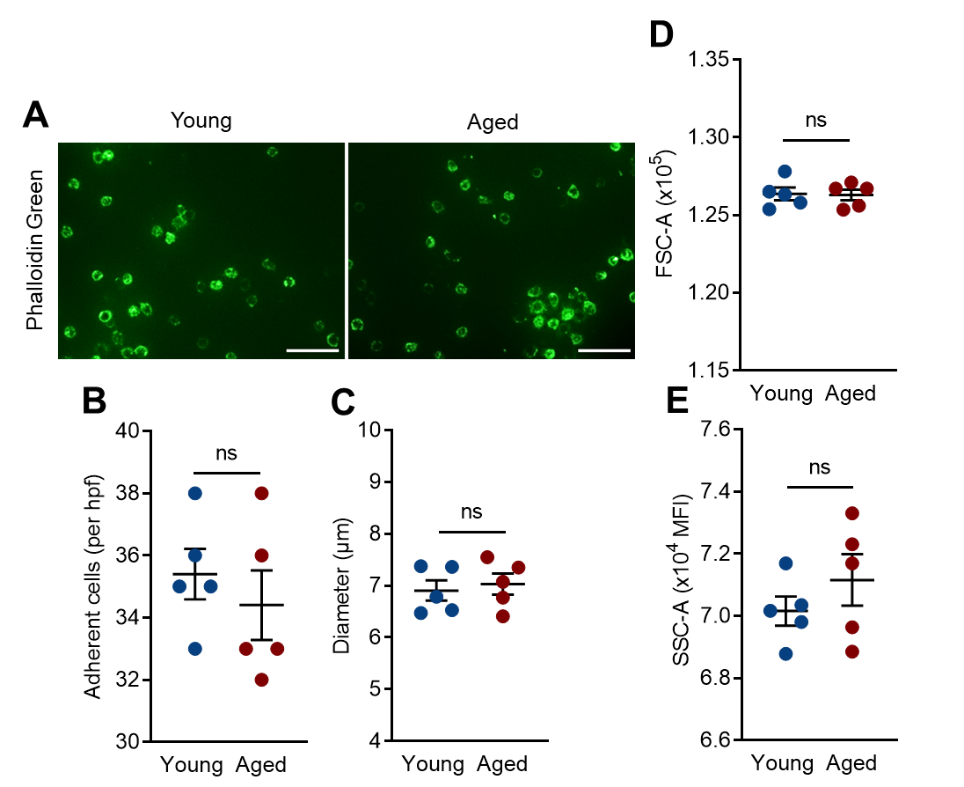


**Supplemental figure 4. Morphological aspect of BM neutrophils**

A-C) The neutrophils were seeded on the coverslip coated with BSA and adherent cells were stained with phalloidin-Alexa 488 to compare the morphology between young and aged mice. A) Representative images of phalloidin staining in neutrophils. B) Number of adherent cells. C) Diameter of adherent neutrophils. D-E) The size and granularity of neutrophils were analyzed by flowcytometry. D) Mean values of FSC-A. E) Mean value of SSC-A. The cumulative data are shown as mean ± SEM values of five samples. Student's t-test was used to analyze data for significant differences. ns; not significant.


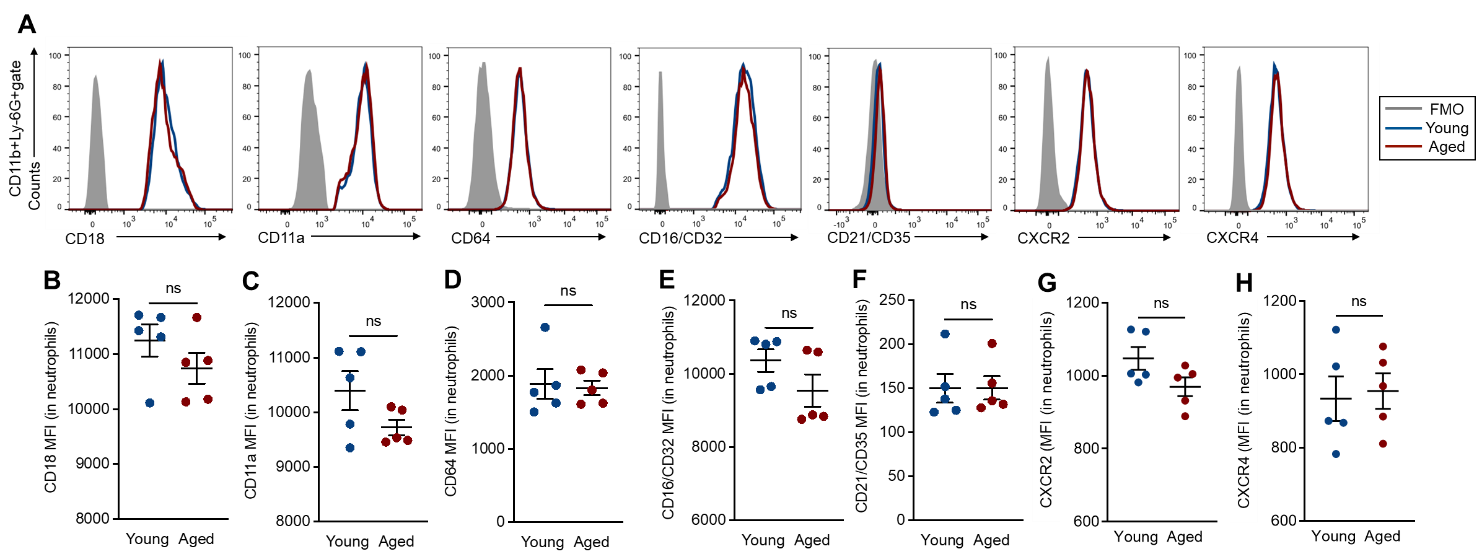


**Supplemental figure 5. Expression intensity of surface markers in BM neutrophils**

Neutrophils were isolated from BM and subjected to analyze the cell surface expression by flow cytometry. A) Representative histogram images of CD18, CD11a, CD64, CD16/CD32, CD21/CD35, CXCR2 and CXCR4. B-H) Cumulative MFI values of CD18 (B), CD11a (C), CD64 (D), CD16/CD32 (E), CD21/CD35 (F), CXCR2 (G) and CXCR4 (H). The cumulative data are shown as mean ± SEM values of five samples. Student's *t*-test was used to analyze data for significant differences. ns; not significant.


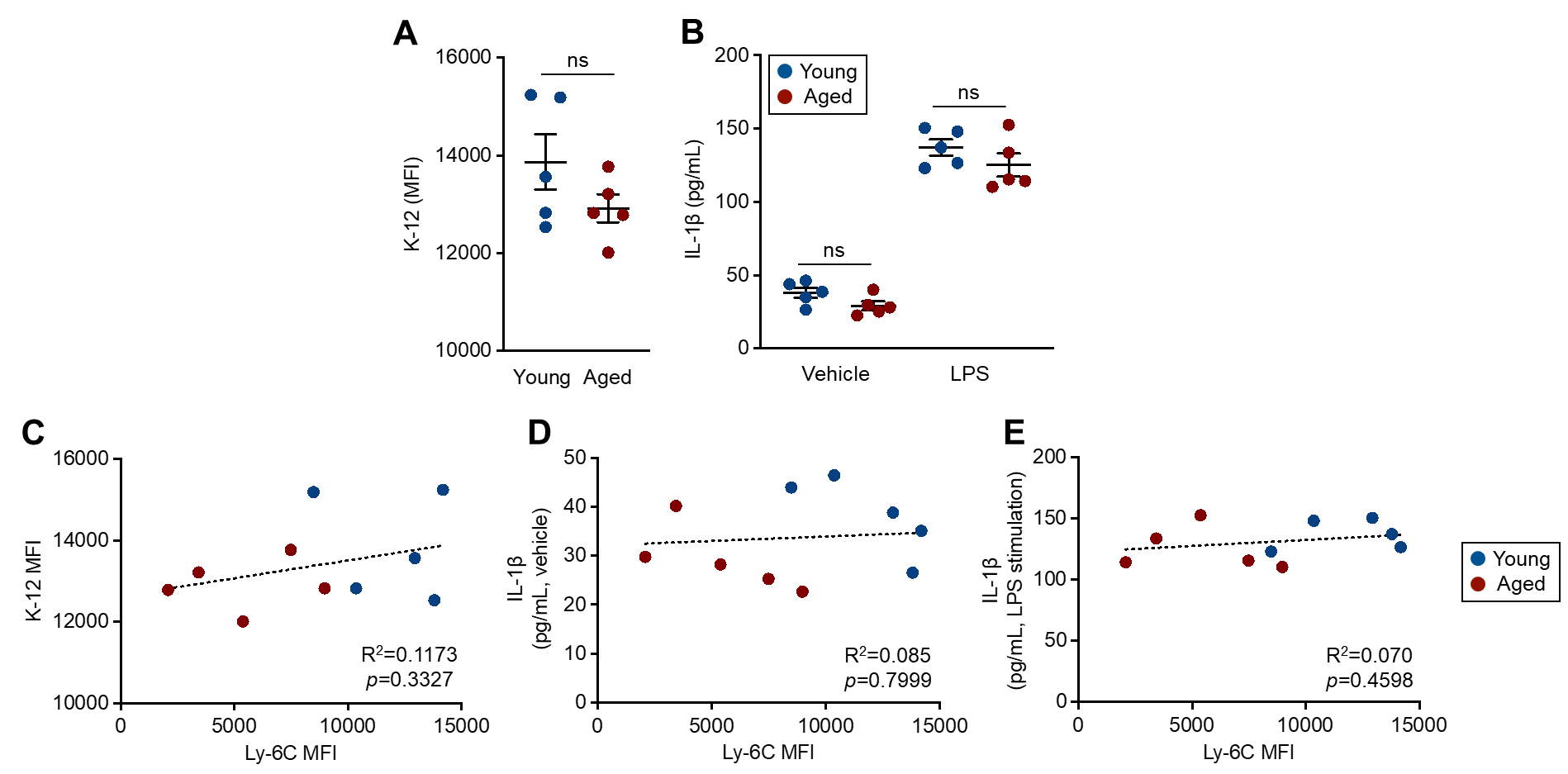


**Supplemental figure 6. Comparison of monocyte function between young and aged mice**

Monocytes were isolated from BM and were subjected to functional assay. A) Phagocytosis assay. The monocytes (1.0x10^7^/mL) were seeded in 96 well plate (round bottom) in RPMI complete medium. The samples were treated with K-12 BioParticle (10 μg/mL) at 37℃ for 120 min. The phagocytosis activity was measured by flow cytometry. B) cytokine production assay. Monocytes (1.0x10^7^/mL) were seeded in 96 well plate (flat bottom), then were stimulated with LPS (1 μg/mL). The control samples were treated with vehicle control (PBS). The cultures were incubated at 37℃ for overnight, then the plate was immediately frozen at -80℃ until use. For measuring the IL-1β concentration in the cultured medium, the plate was centrifuged at 300 *g* for 5 min and supernatant was collected for using ELISA. Correlations between Ly-6C MFI and parameter values gained by functional assays. The linear regression graphs show correlations of Ly-6C MFIs vs the values of incorporated K-12 MFIs (C), IL-1β concentrations in vehicle (D) and LPS (F) treatments, respectively. R^2^ and *p*-values were calculated by using GraphPad prism.
